# 

**Published:** 1899-03-18

**Authors:** 


					* t.,7 ? Dtvrvtrcrrg-
CADBURY'S mcA U CADBURY S
COCOA JOLl J0F& ~ COCOA
ABSOLUTELY PURE. <P> ^ NO DRUGS OR ALKALIES USED.
VoL XXV- [fggg] Saturday,
Maroh 18th, 1899.
[Suta^ripflonT,?..] pEICB TWOpEWCE

				

